# Self-Reported and Physiologic Reactions to Third BNT162b2 mRNA COVID-19 (Booster) Vaccine Dose

**DOI:** 10.3201/eid2807.212330

**Published:** 2022-07

**Authors:** Merav Mofaz, Matan Yechezkel, Grace Guan, Margaret L. Brandeau, Tal Patalon, Sivan Gazit, Dan Yamin, Erez Shmueli

**Affiliations:** Tel Aviv University, Tel Aviv, Israel (M. Mofaz, M. Yechezkel, D. Yamin, E. Shmueli);; Stanford University, Stanford, California, USA (G. Guan, M.L. Brandeau);; Maccabi Healthcare Services, Tel Aviv (T. Patalon, S. Gazit);; Massachusetts Institute of Technology Media Laboratory, Cambridge Massachusetts, USA (E. Shmueli)

**Keywords:** COVID-19, coronavirus disease, severe acute respiratory syndrome coronavirus 2, SARS-CoV-2, viruses, coronaviruses, respiratory infections, vaccine, third COVID-19 dose, booster vaccine, BNT162b2, mRNA, adverse effects, self-reported reactions, heart rate variability, physiologic reactions, wearables, smartwatch, zoonoses

## Abstract

Despite extensive technological advances in recent years, objective and continuous assessment of physiologic measures after vaccination is rarely performed. We conducted a prospective observational study to evaluate short-term self-reported and physiologic reactions to the booster BNT162b2 mRNA (Pfizer-BioNTech, https://www.pfizer.com) vaccine dose. A total of 1,609 participants were equipped with smartwatches and completed daily questionnaires through a dedicated mobile application. The extent of systemic reactions reported after the booster dose was similar to that of the second dose and considerably greater than that of the first dose. Analyses of objective heart rate and heart rate variability measures recorded by smartwatches further supported this finding. Subjective and objective reactions after the booster dose were more apparent in younger participants and in participants who did not have underlying medical conditions. Our findings further support the safety of the booster dose from subjective and objective perspectives and underscore the need for integrating wearables in clinical trials.

The severe acute respiratory syndrome coronavirus 2 Delta variant (also termed variant B.1.617.2) was discovered in October 2020 in India and was designated as a variant of concern by the World Health Organization in May 2021 (*1*–*3*). Since its discovery, it has spread worldwide and has rapidly become the most dominant variant in many countries (*4*–*7*). Although the BNT162b2 COVID-19 vaccine (Pfizer-BioNTech, https://www.pfizer.com) is highly effective against the Alpha variant (*8*), recent studies show that the effectiveness of the Pfizer-BioNTech vaccines is notably lower against the Delta variant: 88% compared with 93.7% against the Alpha variant (*9*–*12*). Moreover, recent evidence shows that fully vaccinated persons infected with the virus can easily transmit it because their peak viral burden is similar to that observed for unvaccinated persons (*7*,*10*). In Israel, the Delta variant has accelerated coronavirus disease (COVID-19) infection and hospitalization; numbers doubled every 10 days during July 1‒August 9, 2021 (*7*,*13*), despite the high coverage of the BNT162b2 vaccine in Israel during this period, which was >75% coverage with 2 Pfizer doses in the eligible population (persons >12 years of age) (*13*).

The rapid increase in hospitalizations associated with the Delta-driven COVID-19 resurgence and the imminent risk for hospital overcrowding led the Israeli government to initialize on July 30, 2021, an unparalleled, proactive, national third (booster) vaccine shot campaign, offering the BNT162b2 mRNA COVID-19 vaccine to persons >60 years of age. On August 13, 2021, the booster campaign was expanded to include persons >50 years of age and reached 63% third-dose coverage among the eligible population within only 26 days (*7*,*14*–*16*). Two weeks later, on August 29, 2021, the campaign was expanded to include all persons >16 years of age, requiring only that 5 months had passed since the receipt of the second dose. This effort reached 40% third-dose coverage among the eligible population <50 years of age within 16 days (*13*,*17*).

Limited information is available on the safety of a BNT162b2 third dose (*18*,*19*). Such a booster vaccine has yet to be authorized by the US Food and Drug Administration (FDA) for the general population (*20*). Although recent evidence shows that a third BNT162b2 dose for immunocompromised persons has a favorable safety profile (*19*,*21*), the safety of a third (booster) dose in the general population has not yet been fully established.

Clinical trial guidelines for assessing the safety of vaccines, including the FDA criteria (*22*), are primarily based on subjective, self-reported questionnaires. Despite the extensive advances in recent years, objective, continuous assessment of physiologic measures postvaccination is rarely performed. Two recent pioneering studies demonstrated the use of wearable devices to monitor short-term physiologic changes after the first and second doses of the BNT162b2 mRNA vaccine. The first study (*23*) used a chest-patch sensor to monitor changes in 13 different cardiovascular and hemodynamic vitals in a cohort of 160 persons up to 3 days postvaccination. The second study (*24*) used a consumer-grade smartwatch to evaluate changes in heart rate variability (HRV), resting heart rate, and respiration rate in a cohort of 19 persons. Both studies found major changes in several physiologic measures in the first days after vaccination.

We evaluated the short-term effects of a third BNT162b2 mRNA COVID-19 vaccine dose on self-reported and physiologic indicators on a relatively large sample. Specifically, we tested 2,912 participants; of these persons, 1,609 participants received >1 doses of the BNT162b2 vaccine after entering the study. Participants were equipped with Garmin (https://www.garmin.com) Vivosmart 4 smart fitness trackers and completed daily questionnaires by using a dedicated mobile application for 37 days, starting 7 days before vaccination. The mobile application collected daily self-reported questionnaires on local and systemic reactions, as well as various well-being indicators. The smartwatch continuously monitored several physiologic measures, including heart rate, HRV, and blood oxygen saturation level (SpO_2_). Our analysis of comprehensive data for each participant examined the safety of a third (booster) vaccine dose from a subjective perspective (self-reported questionnaire) and an objective perspective (smartwatch data).

## Materials and Methods

### Study Design and Participants

The 2,912 participants (>18 years of age) in our study were recruited during November 1, 2020‒September 15, 2021. The 1,609 participants who reported receipt of >1 of the 3 BNT162b2 mRNA COVID-19 vaccine shots after joining the study served as the base dataset for our analysis. All participants received the BNT162b2 mRNA vaccine. Specifically, of the 1,609 participants, during the study, 223 received their first dose, 351 their second dose, and 1,344 their third dose. Among these participants, 111 received both the second and third doses, 85 received both the first and third doses, and 80 received all 3 doses.

We used a professional survey company to recruit participants and ensure they followed through with the study requirements. Participant recruitment was performed by using advertisements on social media and word-of-mouth. Each participant provided informed consent by signing a form after receiving a comprehensive explanation on the study. Participants then completed a 1-time enrollment questionnaire, were equipped with Garmin Vivosmart 4 smartwatches, and installed 2 applications on their mobile phones: the PerMed application (*25*), which collected daily self-reported questionnaires, and an application that passively recorded the smartwatch data. Participants were asked to wear their smartwatches as much as possible. The survey company ensured that participants’ questionnaires were completed daily, that their smartwatches were charged and properly worn, and that any technical problems with the mobile applications or smartwatch were resolved. Participants were monitored through the mobile application and smartwatches for 37 days, starting 7 days before vaccination.

We implemented several preventive measures to minimize attrition and churn (attrition rate) of participants and consequently improve the quality, continuity, and reliability of the collected data. First, each day, if by 7:00 pm participants had not yet completed the daily questionnaire, they received a reminder notification through the PerMed application. During the peak periods of COVID-19 vaccination in Israel, we increased the frequency of the reminders and adjusted their content. Second, we developed a dedicated dashboard that enabled the survey company to identify participants who continually neglected to complete the daily questionnaires or did not wear their smartwatch for a long period of time; these participants were contacted by the survey company (either by text messages or telephone calls) and were encouraged to better adhere to the study protocol. Third, to strengthen participants’ engagement, a weekly personalized summary report was generated for each participant and was available inside the PerMed application. Similarly, we sent a monthly newsletter that contained recent findings from the study and useful tips regarding the smartwatch’s capabilities to the participants.

### PerMed Mobile Application

Participants used the PerMed mobile application (*25*) to fill out daily questionnaires. The questionnaire enabled participants to report various well-being indicators, including mood level (on a scale of 1 [awful] to 5 [excellent]), stress level (on a scale of 1 [very low] to 5 [very high]), sport activity duration (in minutes), and sleep quality (on a scale of 1 [awful] to 5 [excellent]). The questionnaire also collected data on clinical symptoms consistent with the local and systemic reactions observed in the BNT162b2 mRNA COVID-19 clinical trial (*26*), with an option to add other symptoms as free text (Appendix).

### Smartwatch

Participants were equipped with Garmin Vivosmart 4 smart fitness trackers. Among other features, the smartwatch provides all-day heart rate and HRV and overnight SpO_2_ tracking capabilities (*27*).

The optical wrist heart rate monitor of the smartwatch is designed to continuously monitor heart rate. The frequency at which heart rate is measured varies and might depend on the level of activity of the user: when the user starts an activity, the optical heart rate monitor’s measurement frequency increases.

Because HRV is not easily accessible through Garmin’s application programming interface, we use Garmin’s stress level instead, which is calculated on the basis of HRV. Specifically, the device uses heart rate data to determine the interval between each heartbeat. The variable length of time between each heartbeat is regulated by the body’s autonomic nervous system. Less variability between beats correlates with higher stress levels, whereas an increase in variability indicates less stress (*28*). A similar relationship between HRV and stress was also seen by Kim et al. (*29*) and Pereira et al. (*30*).

The pulse oximetry monitor of the smartwatch uses a combination of red and infrared lights with sensors on the back of the device to estimate the percentage of oxygenated blood (peripheral SpO_2_%). This monitor is activated each day at a fixed time for 4 hours (the default is 2:00‒6:00 am). When we examined data collected in our study, we identified a heart rate sample approximately every 15 seconds, an HRV sample every 180 seconds, and an SpO_2_ sample every 60 seconds.

Although the Garmin smartwatch provides state-of-the-art wrist monitoring, it is not a medical-grade device. Some readings might be inaccurate under certain circumstances, depending on factors such as the fit of the device and the type and intensity of the activity undertaken by a participant (*31*–*33*).

### Statistical Analysis

We preprocessed questionnaire data by manually categorizing any self-reported symptom entered as free text. In addition, if participants completed the questionnaire >1 time in 1 day, we used the last entry from that day for the analysis. We preprocessed smartwatch data as follows. We computed the mean value of each hour of data. We then performed linear interpolation to impute missing hourly means and smoothed the data by calculating the 5-hour moving average.

For each participant, we defined the 7-day period before vaccination as the baseline period. We noted any clinical symptoms from the last questionnaire completed during the baseline period. Next, we calculated the percentage of participants who reported new systemic reactions in the 48 hours after vaccination. For each reaction, we used a β distribution to determine a 90% CI. To determine the statistical significance of differences between the first and third doses and between the second and third doses as reflected by the extent of reported reactions, we used a test for comparing proportions of 2 partially overlapping samples with unequal variance (*34*).

We also calculated the mean difference in well-being indicators between the postvaccination and baseline periods. Specifically, for each indicator, for each of the 3 days postvaccination and for each participant, we calculated the difference between that indicator’s value and its corresponding value in the baseline period. We then calculated the mean value over all participants and the associated 90% CI.

To compare the changes in smartwatch physiologic indicators over the 7 days (168 hours) postvaccination with those of the baseline period, we performed the following steps. First, for each participant and each hour during the 7 days postvaccination, we calculated the difference between that hour’s indicator value and that of the corresponding hour in the baseline period (keeping the same day of the week and same hour during the day). Then, we aggregated each hour’s differences over all participants to calculate a mean difference and associated 90% CI, which is analogous to a 1-sided t-test a with significance level of 0.05. To determine the statistical significance of differences between the first and third doses and between the second and third doses as reflected by changes in smartwatch indicators during the 48 hours postvaccination, we used a test for comparing means of 2 partially overlapping samples with unequal variance (*35*).

We repeated our analyses for the third dose stratified by age groups (<50, 50–64, and >65 of age), sex, and underlying medical condition (present versus not present) from a specified list (Table). To determine the statistical significance of differences between the groups in these analyses, we used a t-test for comparing the means of 2 independent samples with unequal variance.

**Table:** Characteristics of participants in study of self-reported and physiologic reactions to third BNT162b2 mRNA coronavirus disease (booster) vaccine dose*

Characteristic	All participants, n = 1,609	First dose, n = 223	Second dose, n = 351	Third dose, n = 1,344
Sex				
M	755 (46.92)	101 (45.29)	160 (45.58)	639 (47.54)
F	854 (53.08)	122 (54.71)	191 (54.42)	705 (52.46)
Age group, y				
18–29	226 (14.23)	14 (6.28)	39 (11.11)	189 (14.06)
30–39	272 (16.90)	11 (4.93)	53 (15.10)	219 (16.29)
40–49	177 (11.00)	15 (6.73)	42 (11.97)	138 (10.27)
50–59	420 (26.10)	64 (28.70)	87 (24.79)	375 (27.90)
60–69	358 (22.25)	70 (31.39)	75 (21.37)	308 (22.92)
>70	153 (9.51)	49 (21.97)	55 (15.67)	8.56 (115)
Body mass index, kg/m^2^				
<30.0	1,258 (78.19)	175 (78.48)	280 (79.77)	77.68 (1,044)
>30.0	330 (20.51)	41 (18.39)	60 (17.09)	288 (21.43)
Unspecified	21 (1.31)	7 (3.14)	11 (3.13)	12 (0.89)
Underlying medical condition				
Hypertension	228 (14.17)	20.63 (46)	15.95 (56)	14.43 (194)
Diabetes	139 (8.64)	13.00 (29)	7.98 (28)	8.41 (113)
Heart disease	77 (4.79)	7.17 (16)	4.56 (16)	4.99 (67)
Chronic lung disease	81 (5.03)	4.93 (11)	3.70 (13)	5.21 (70)
Immune suppression	13 (0.81)	1.35 (3)	0.85 (3)	0.89 (12)
Cancer	10 (0.62)	0.45 (1)	0.57 (2)	0.67 (9)
Renal failure	8 (0.50)	1.79 (4)	1.42 (5)	0.45 (6)
None of the above	1,180 (73.34)	64.57 (144)	72.08 (253)	73.21 (984)
Unspecified	17 (1.06)	1.35 (3)	2.85 (10)	0.52 (7)

*Values are no. (%). BNT162b2 vaccine, Pfizer-BioNTech (https://www.pfizer.com).

### Ethics Approval

Before participating in the study, all persons were advised, both orally and in writing, as to the nature of the study and provided written informed consent. The study was approved by the Maccabi Health Services Helsinki Institutional Review Board (protocol no. 0122–20-MHS).

## Results

Of the 1,609 participants who received >1 dose of the BNT162b2 vaccine after joining the study, 854 (53.08%) were women and 755 (46.92%) men. Their ages were 18‒88 years; median age was 52 years (Table). A total of 1,258 (78.19%) participants had a body mass index <30 kg/m^2^, and 412 (25.61%) had >1 specific underlying medical condition (Table). The distributions of age and sex and underlying medical conditions were relatively invariable across the recipients of the first, second, and third doses (Table).

**Figure 1:**
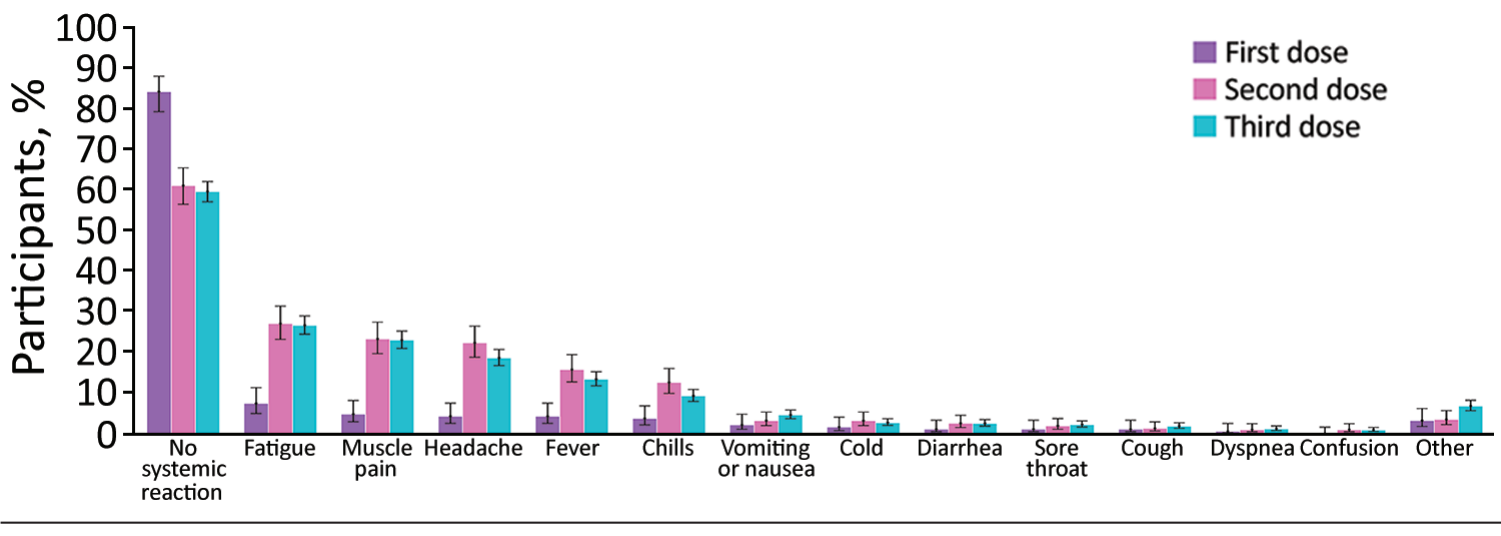
Reactions reported by participants through a mobile application for self-reported and physiologic reactions to BNT162b2 (Pfizer, https://www.pfizer.com) mRNA coronavirus disease vaccine doses. Error bars indicate 90% CIs.

Our examination of self-reported reactions showed that the extent of systemic reactions reported after the third vaccine dose was similar to those reported after the second dose (p = 0.76) and considerably greater than those observed after the first dose (p<0.001) (Figure 1). Specifically, 60.4% (90% CI 57.9%–62.9%) of the participants did not report any new symptoms after receiving the third dose compared with 86.5% (90% CI 81.9%–91.0%) after the first dose and 63.6% (90% CI 59.1%–67.8%) after the second dose. Moreover, the most frequently reported types of reactions (fatigue, headache, muscle pain, fever, and chills) were similar after the second and third doses. These reactions decreased in nearly all participants within 3 days (Appendix Figure 8). These trends are consistent with those reported for the first and second dose BNT162b2 mRNA vaccine clinical trial (*26*).

**Figure 2:**
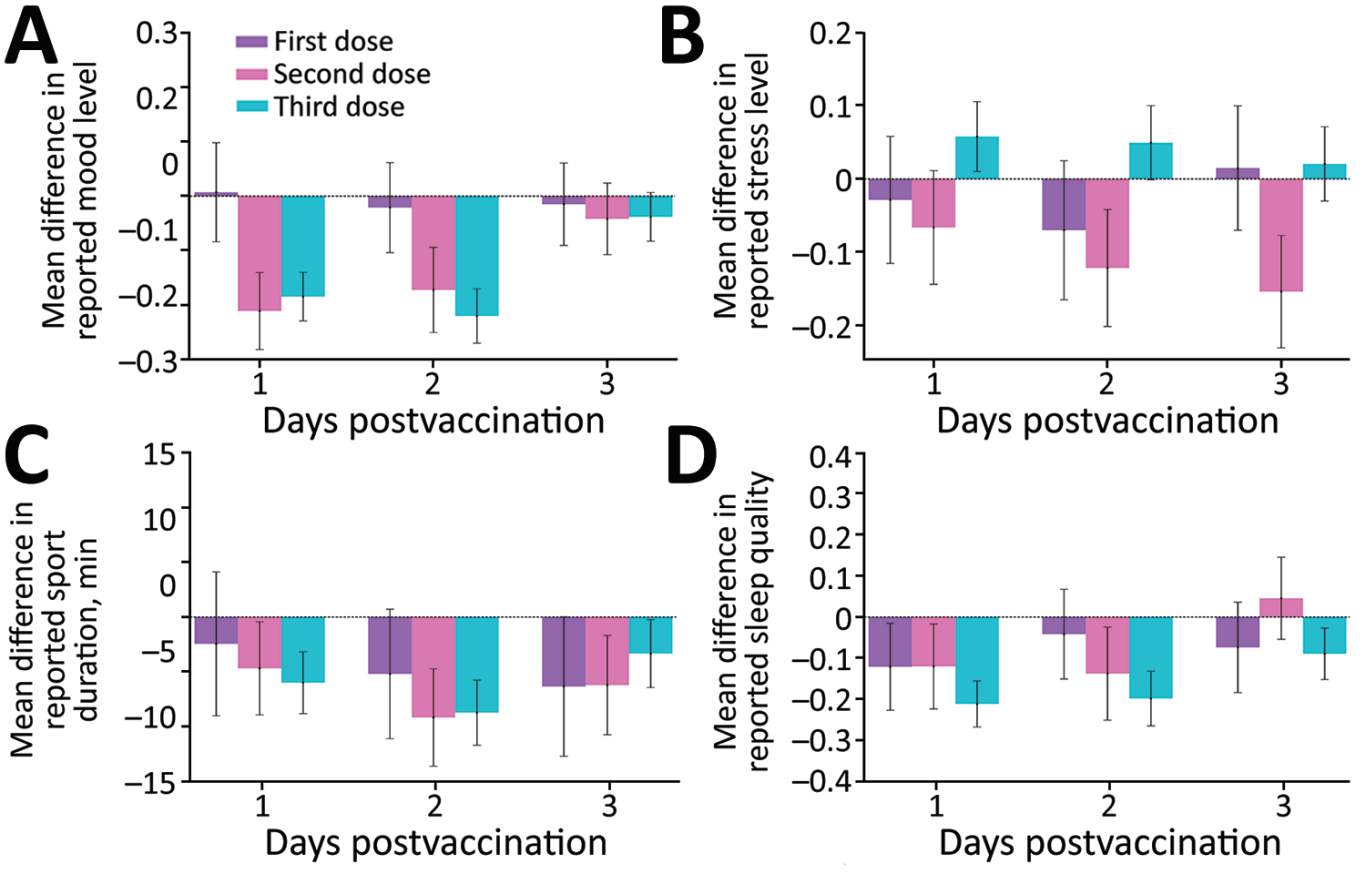
Changes in subjective well-being indicators reported by participants through a mobile application for self-reported and physiologic reactions to BNT162b2 (Pfizer, https://www.pfizer.com) mRNA coronavirus disease vaccine doses. Mean difference compared with baseline levels are shown for the well-being indicators of mood level (A), stress level (B), sport duration (C), and sleep quality (D). Mood level, stress level, and sleep quality were reported on a 1–5 Likert scale. Sport duration was measured in minutes. Error bars indicate 90% CIs. Horizontal dashed lines indicate no change compared with baseline levels.

For the self-reported well-being indicators (Figure 2), we found that during the first 2 days after the third vaccine dose, participants showed a major reduction in mood level (Figure 2, panel A), sport duration (Figure 2, panel C), and sleep quality (Figure 2, panel D) and a large increase in stress level (Figure 2, panel B) compared with baseline levels. These changes decreased on the third day postvaccination. A similar trend was observed after the second vaccine dose, except for the reported stress level, which remained below the baseline level during the second and third days postvaccination.

**Figure 3:**
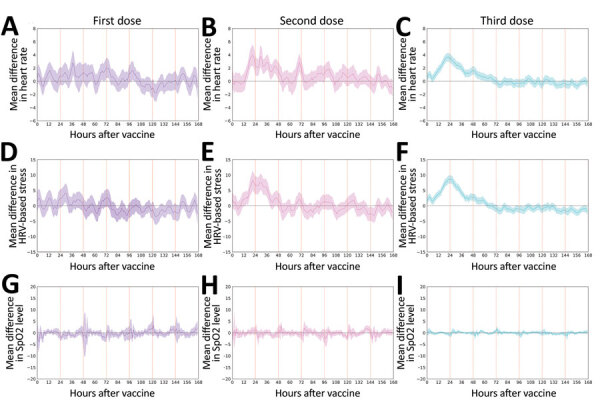
Changes in objective physiologic indicators measured through smartwatch for self-reported and physiologic reactions to BNT162b2 (Pfizer, https://www.pfizer.com) mRNA coronavirus disease vaccine doses. Mean difference are shown for smartwatch-recorded heart rate (A‒C), HRV-based stress (D‒F), and SpO_2_ (G‒I) after the first, second, and third dose compared with their baseline levels. Mean values are indicated by solid lines; 90% CIs are indicated as shaded regions. Horizontal dashed line indicates no change compared with baseline levels, and vertical lines indicate 24-hour periods. HRV, heart-related variability; SpO_2_, blood oxygen saturation level.

We observed similar trends when analyzing objective and continuous physiologic measurements collected by the smartwatch (Figure 3; Appendix Figure 1). Specifically, we identified a considerable increase in heart rate (Figure 3, panels A‒C) and the HRV-based stress indicators (Figure 3, panels D‒F) during the first 48 hours after administration of the third dose. Measurements returned to baseline levels within 72 hours. In contrast, our analysis of SpO_2_ suggests no apparent changes after vaccination compared with baseline levels (Figure 3, panels G‒I), a result that is consistent with the results of Gepner et al. (*23*). The trends observed for the objective heart rate and HRV indicators were consistent with those of the subjective indicators: similar changes after the second and third doses (heart rate p = 0.86, HRV p = 0.54), and greater changes after the third dose than the first dose (heart rate p = 0.004, HRV p<0.001).

**Figure 4:**
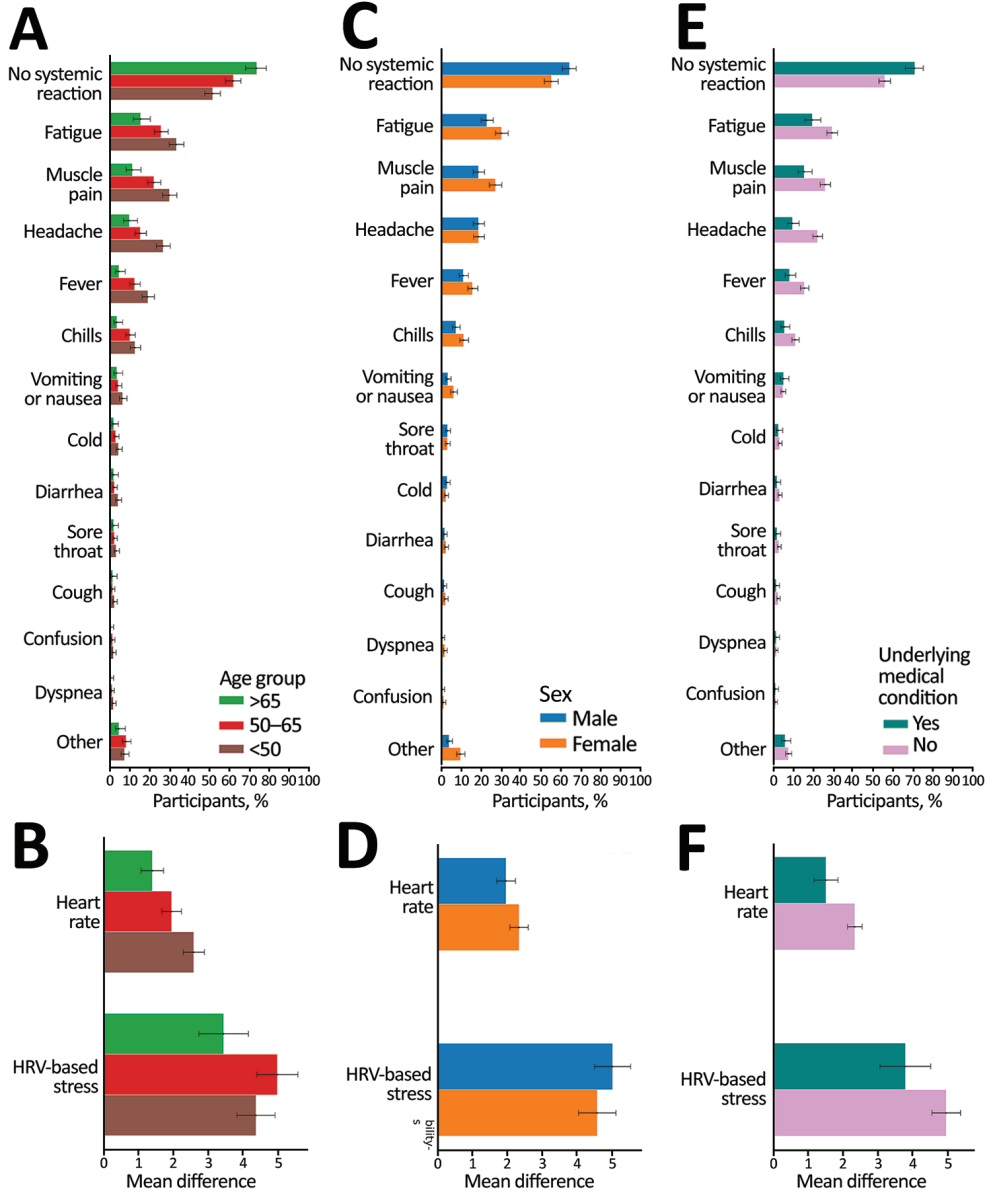
Self-reported and objective reactions following the third vaccine dose, stratified by age, sex, and underlying medical condition for self-reported and physiologic reactions to third BNT162b2 (Pfizer, https://www.pfizer.com) mRNA coronavirus disease vaccine doses. Reactions reported by participants through the mobile application (A, C, E) and objective heart rate and heart rate variability measured through a smartwatch (B, D, F) are shown, stratified by age (A, B), sex (C, D), and underlying medical condition (E, F). Bars indicate percentage of participants with a reported or recorded reaction; error bars indicate 90% CIs.

We also stratified our analyses of well-being and smartwatch physiologic indicators after the third vaccination by age group, sex, and a previous underlying medical condition (Figure 4; Appendix Figures 2‒7). For all stratifications, trends were similar to those observed in the general population. We found considerable changes in the 2 days after vaccine administration that decreased almost entirely after 3 days. We also found that participants >65 years of age reported fewer reactions (p<0.001) than did participants 50–65 years of age, who in turn reported even fewer reactions (p = 0.007) than did participants <50 years of age (Figure 4, panel A). In terms of the objective physiologic measures, participants >65 years of age showed milder changes in HRV than did participants 50–65 years of age (p = 0.075) and milder changes in heart rate (p = 0.02) than did participants <50 years of age (Figure 4, panel B). 

Male participants reported fewer reactions (p<0.001) but did not show milder physiologic changes (heart rate p = 0.37, HRV p = 0.59) than female participants. Participants who had an underlying medical condition reported fewer reactions (p<0.001) and showed milder physiologic changes (heart rate p = 0.042, HRV p = 0.16), compared with participants who did not have an underlying medical condition. Of 9 participants who reported dyspnea, 4 (0.96% of their age group) were <50 years of age, 4 (0.93% of their age group) were 50–64 years of age, and 1 (0.65% of her age group) was >65 years of age. One participant <50 years of age reported chest pain after vaccination. None of these participants had an underlying medical condition. These reactions (i.e., dyspnea and chest pain) disappeared 2–4 days after vaccination.

## Discussion

Our key findings suggest that local and systemic reactions reported after the third (booster) vaccine dose administration are similar to those reported after the second dose and considerably greater than those observed after the first dose. Our analyses of self-reported well-being indicators and objective smartwatch physiologic indicators underscore these results. Furthermore, within 3 days from vaccination with the third dose, all measures returned to their baseline levels in all participants. We identified differences in subpopulations on the basis of sex, age, and underlying medical conditions after administration of the third vaccine dose. It has been suggested that reactions caused by the COVID-19 vaccine are a byproduct of a short burst of interferon type I generation concomitant with induction of an effective immune response (*36*). Interferon type I generation is substantially stronger in women than in men and stronger in younger and healthier persons than in older and less healthy persons. We found that participants <65 years of age, female participants, and participants without an underlying medical condition showed greater reactions in self-reported local and systemic reactions and well-being indicators, as well as in objective physiologic measurements recorded by the smartwatch. Our results are also consistent with the results of a previous study that found similar trends after the first and second doses (*37*).

Clinical trials have not yet used the comprehensive physiologic measures generated by wearable devices, such as smartwatches. Currently, the FDA and European Medicines Agency evaluate the safety of and create guidelines for newly developed vaccines primarily on the basis of subjective, self-reported questionnaires (*22*,*38*). Much of the scientific literature discusses these self-reported side effects of COVID-19 vaccines. However, integrating wearable devices into clinical trials, alongside self-reported questionnaires, can provide more precise and rich data regarding the vaccines’ effects on physiologic measures.

Our study’s first limitation is that the 1,609 persons who comprised the base dataset of our analyses might not be representative of the vaccinated population in Israel or globally. Nevertheless, the changes observed in self-reported reactions and well-being indicators, as well as objective physiologic indicators recorded by the smartwatches, were statistically significant and consistent with each other. Moreover, the reaction types, frequency, and duration we observed for the first and second doses were similar to those observed in the BNT162b2 mRNA vaccine clinical trials (*26*). In addition, a clear pattern of returning to baseline levels was observed within 72 hours after vaccination in all examined measures. Although the sample size was limited, trends were consistent regardless of age group, sex, and underlying medical conditions.

Second, we did not explicitly control for the effects of the observational trial setting (e.g., participating in a trial, wearing a smartwatch, potential concerns regarding the vaccine). Any effects of the observational trial setting should, in principle, have similar effects on our analysis of each of the 3 vaccine doses. However, because we found no deviations in most measurements from baseline levels in the subset of participants who received their first dose, we believe the changes observed after the second and third doses arise from an actual reaction to the vaccine.

Third, the smartwatches used to obtain physiologic measurements are not medical-grade devices. Nevertheless, recent studies show a considerably accurate heart rate measurement in the previous versions of the smartwatch used in this study (*31*,*32*). In the same context, for some measures, such as SpO_2_, the timing of measurement might be different across participants (e.g., if they changed their default settings). In both instances, it is useful to emphasize that our analyses focused on the change in measurements compared with their baseline values, rather than on their absolute values.

Fourth, all participants in our study received the BNT162b2 mRNA vaccine. Although our findings might not be directly generalized to other types of COVID-19 vaccines, we believe that applying our analyses on other vaccines is likely to yield qualitatively similar findings because of the similarities observed between different COVID-19 vaccines (*26*,*39*,*40*).

It would be useful to evaluate the effect of previous COVID-19 infection episodes on the results we obtained. However, although our data set contains some information on COVID-19 infections of participants during the time they spent in the study, it lacks information on infection episodes that occurred before they joined the study, making such analyses an interesting topic for future research.

Our study strengthens the evidence regarding the short-term safety of the booster BNT162b2 vaccine in several ways. First, reports of local and systemic reactions after the third dose were similar to those observed after the second dose, which was shown in clinical trials to be safe (*26*). Second, the considerable changes observed for all indicators during the first 2 days after receiving the third vaccine, including self-reported reactions and well-being indicators, as wells as objective physiologic indicators collected by the smartwatch, returned to their baseline levels. Third, regardless of the observed differences between subpopulations, our analyses indicated a clear pattern of return to baseline levels in all considered subpopulations. Fourth, we observed no change in SpO_2_ compared with baseline levels, indicating that major adverse health consequences are less likely.

In conclusion, our study supports the short-term safety of the third BNT162b2 mRNA COVID-19 (booster) vaccine dose and mitigates, in part, concerns regarding its short-term effects. The medical and scientific communities could greatly benefit from the largely unbiased data generated by digital health technologies, such as the wearable devices that we analyzed in this study. Our findings could also be of interest to public health officials and other stakeholders because it is essential that objective measures are given attention in the critical evaluation of clinical trials.

Appendix Additional information on self-reported and physiologic reactions to third BNT162b2 mRNA COVID-19 (booster) vaccine dose.

## References

[1] Kupferschmidt K, Wadman M. Delta variant triggers new phase in the pandemic. Science. 2021;372:1375–6. 10.1126/science.372.6549.1375

[2] World Health Organization. Tracking SARS-CoV-2 variants [cited 2022 Apr 13]. https://www.who.int/en/activities/tracking-SARS-CoV-2-variants 37184162

[3] Mlcochova P, Kemp SA, Dhar MS, Papa G, Meng B, Ferreira IATM, et al.; Indian SARS-CoV-2 Genomics Consortium (INSACOG); Genotype to Phenotype Japan (G2P-Japan) Consortium; CITIID-NIHR BioResource COVID-19 Collaboration. SARS-CoV-2 B.1.617.2 Delta variant replication and immune evasion. Nature. 2021;599:114–9. 10.1038/s41586-021-03944-y 34488225 PMC8566220

[4] Centers for Disease Control and Prevention. Delta variant: what we know about the science [cited 2022 Apr 13]. https://stacks.cdc.gov/view/cdc/108671

[5] Mahase E. Delta variant: What is happening with transmission, hospital admissions, and restrictions? BMJ. 2021;373:n1513. 10.1136/bmj.n1513 34130949

[6] World Health Organization. Weekly epidemiological update on COVID-19, August 17, 2021 [cited 2022 Apr 13]. https://www.who.int/publications/m/item/weekly-epidemiological-update-on-covid-19---17-august-2021

[7] Wadman M. Israel’s grim warning: Delta can overwhelm shots. Science. 2021;373:838–9. 10.1126/science.373.6557.838 34413215

[8] Munitz A, Yechezkel M, Dickstein Y, Yamin D, Gerlic M. BNT162b2 vaccination effectively prevents the rapid rise of SARS-CoV-2 variant B.1.1.7 in high-risk populations in Israel. Cell Rep Med. 2021;2:100264. 10.1016/j.xcrm.2021.100264 33899031 PMC8053239

[9] Lopez Bernal J, Andrews N, Gower C, Gallagher E, Simmons R, Thelwall S, et al. Effectiveness of COVID-19 vaccines against the B.1.617.2 (Delta) variant. N Engl J Med. 2021;385:585–94. 10.1056/NEJMoa2108891 34289274 PMC8314739

[10] Pouwels KB, Pritchard E, Matthews PC, Stoesser N, Eyre DW, Vihta K-D, et al. Effect of Delta variant on viral burden and vaccine effectiveness against new SARS-CoV-2 infections in the UK. Nat Med. 2021;27:2127–35. 10.1038/s41591-021-01548-7 34650248 PMC8674129

[11] Sheikh A, McMenamin J, Taylor B, Robertson C; Public Health Scotland and the EAVE II Collaborators. SARS-CoV-2 Delta VOC in Scotland: demographics, risk of hospital admission, and vaccine effectiveness. Lancet. 2021;397:2461–2. 10.1016/S0140-6736(21)01358-1 34139198 PMC8201647

[12] Nasreen S, He S, Chung H, Brown KA, Gubbay JB, Buchan SA, et al. Effectiveness of COVID-19 vaccines against variants of concern in Ontario. Nat Microbiol. 2022;7:379–85. 10.1038/s41564-021-01053-0 35132198

[13] Centers for Disease Control and Prevention. COVID-19 datasets—government data. [cited 2022 Apr 13]. https://data.cdc.gov/browse?q=covid-19

[14] Ministry of Health. The Ministry of Health Director General has approved the recommendation to administer a third vaccine dose to 50-year-olds and older and to other population [cited 2022 Apr 13]. https://www.gov.il/en/departments/news/13082021-01

[15] Ministry of Health. The Vaccination Advisory Committee presented data and recommended the administration of a third dose to older adults [cited 2022 Apr 13]. https://www.gov.il/en/departments/news/29072021-04

[16] Prime Minister’s Office. Statement by PM Bennett on third dose of the COVID vaccine to Israeli citizens over the age of 60 [cited 2022 Apr 13]. https://www.gov.il/en/Departments/news/event_press29071

[17] Ministry of Health. The vaccination policy and the new green pass that will take effect soon [cited 2022 Apr 13]. https://www.gov.il/en/departments/news/29082021-01

[18] Centers for Disease Control and Prevention. Joint statement from HHS public health and medical experts on COVID-19 booster shots. CDC online newsroom [2022 Apr 13]. https://www.cdc.gov/coronavirus/2019-ncov/vaccines/booster-shot.html?s_cid=11709:covid%20vaccine%20booster:sem.ga:p:RG:GM:gen:PTN.Grants:FY22

[19] Centers for Disease Control and Prevention. COVID-19 vaccines for people who are moderately to severely immunocompromised [cited 2022 Apr 13]. https://www.cdc.gov/coronavirus/2019-ncov/vaccines/recommendations/immuno.html?s_cid=10483:immunocompromised%20and%20covid%20vaccine:sem.ga:p: GM:gen:PTN:FY21

[20] US Food and Drug Administration. Joint statement from HHS public health and medical experts on COVID-19 booster shots [2022 Apr 13]. https://www.hhs.gov/about/news/2021/08/18/joint-statement-hhs-public-health-and-medical-experts-covid-19-booster-shots.html

[21] Kamar N, Abravanel F, Marion O, Couat C, Izopet J, Del Bello A. Three doses of an mRNA Covid-19 vaccine in solid-organ transplant recipients. N Engl J Med. 2021;385:661–2. 10.1056/NEJMc2108861 34161700 PMC8262620

[22] Food and Drug Administration. Development and licensure of vaccines to prevent COVID-19 [cited 2022 Apr 13]. https://www.fda.gov/media/139638/download

[23] Gepner Y, Mofaz M, Oved S, Yechezkel M, Constantini K, Goldstein N, et al. Utilizing wearable sensors for continuous and highly-sensitive monitoring of reactions to the BNT162b2 mRNA COVID-19 vaccine. Commun Med (Lond). 2022;2:27. 10.1038/s43856-022-00090-y 35603274 PMC9053261

[24] Hajduczok AG, DiJoseph KM, Bent B, Thorp AK, Mullholand JB, MacKay SA, et al. Physiologic response to the Pfizer-BioNTech COVID-19 vaccine measured using wearable devices: prospective observational study. JMIR Form Res. 2021;5:e28568. 10.2196/28568 34236995 PMC8341091

[25] Oved S, Mofaz M, Lan A, Einat H, Kronfeld-Schor N, Yamin D, et al. Differential effects of COVID-19 lockdowns on well-being: interaction between age, gender and chronotype. J R Soc Interface. 2021;18:20210078. 10.1098/rsif.2021.0078 34062107 PMC8169206

[26] Polack FP, Thomas SJ, Kitchin N, Absalon J, Gurtman A, Lockhart S, et al.; C4591001 Clinical Trial Group. C4591001 Clinical Trial Group. Safety and efficacy of the BNT162b2 mRNA COVID-19 vaccine. N Engl J Med. 2020;383:2603–15. 10.1056/NEJMoa2034577 33301246 PMC7745181

[27] Garmin. VÍVOSMART® 4 owner’s manual. 2018 [cited 2022 Apr 13]. https://www8.garmin.com/manuals/webhelp/vivosmart4/EN-US/vivosmart_4_OM_EN-US.pdf

[28] Garmin. What is the stress level feature on my Garmin watch? [cited 2022 Apr 13]. https://support.garmin.com

[29] Kim H-G, Cheon E-J, Bai D-S, Lee YH, Koo B-H. Stress and heart rate variability: a meta-analysis and review of the literature. Psychiatry Investig. 2018;15:235–45. 10.30773/pi.2017.08.17 29486547 PMC5900369

[30] Pereira T, Almeida PR, Cunha JPS, Aguiar A. Heart rate variability metrics for fine-grained stress level assessment. Comput Methods Programs Biomed. 2017;148:71–80. 10.1016/j.cmpb.2017.06.018 28774440

[31] Reddy RK, Pooni R, Zaharieva DP, Senf B, El Youssef J, Dassau E, et al. Accuracy of wrist-worn activity monitors during common daily physical activities and types of structured exercise: evaluation study. JMIR Mhealth Uhealth. 2018;6:e10338. 10.2196/10338 30530451 PMC6305876

[32] Bent B, Goldstein BA, Kibbe WA, Dunn JP. Investigating sources of inaccuracy in wearable optical heart rate sensors. NPJ Digit Med. 2020;3:18. 10.1038/s41746-020-0226-6 32047863 PMC7010823

[33] Garmin. Accuracy [cited 2022 Apr 13]. https://support.garmin.com/en-US/?faq=aZc8RezeAb9LjCDpJplTY7#:~:text=Garmin%C2%AE%20GPS%20receivers%20are,to%20its%20Satellite%20Information%20Page

[34] Derrick B, Dobson-Mckittrick A, Toher D, White P. Test statistics for comparing two proportions with partially overlapping samples. J Appl Quant Methods. 2015;10:1–14.

[35] Derrick B, Russ B, Toher D, White P. Test statistics for the comparison of means for two samples that include both paired and independent observations. J Mod Appl Stat Methods. 2017;16:9. 10.22237/jmasm/1493597280

[36] Bunders MJ, Altfeld M. Implications of sex differences in immunity for SARS-CoV-2 pathogenesis and design of therapeutic interventions. Immunity. 2020;53:487–95. 10.1016/j.immuni.2020.08.003 32853545 PMC7430299

[37] Menni C, Klaser K, May A, Polidori L, Capdevila J, Louca P, et al. Vaccine side-effects and SARS-CoV-2 infection after vaccination in users of the COVID Symptom Study app in the UK: a prospective observational study. Lancet Infect Dis. 2021;21:939–49. 10.1016/S1473-3099(21)00224-3 33930320 PMC8078878

[38] European Medicines Agency. Clinical trials in human medicines [cited 2022 Apr 13]. https://www.ema.europa.eu/en/human-regulatory/research-development/clinical-trials-human-medicines

[39] Baden LR, El Sahly HM, Essink B, Kotloff K, Frey S, Novak R, et al.; COVE Study Group. Efficacy and safety of the mRNA-1273 SARS-CoV-2 vaccine. N Engl J Med. 2021;384:403–16. 10.1056/NEJMoa2035389 33378609 PMC7787219

[40] Voysey M, Clemens SAC, Madhi SA, Weckx LY, Folegatti PM, Aley PK, et al.; Oxford COVID Vaccine Trial Group. Safety and efficacy of the ChAdOx1 nCoV-19 vaccine (AZD1222) against SARS-CoV-2: an interim analysis of four randomised controlled trials in Brazil, South Africa, and the UK. Lancet. 2021;397:99–111. 10.1016/S0140-6736(20)32661-1 33306989 PMC7723445

