# Supplementary material for: Self-Reported and Physiologic Reactions to Third BNT162b2 mRNA COVID-19 (Booster) Vaccine Dose
Source: Emerg Infect Dis. 2022 Jul;28(7):1375–83. doi: 10.3201/eid2807.212330 (PMC9239876; doi:10.3201/eid2807.212330)
Supplement: Appendix — Additional information on self-reported and physiologic reactions to third BNT162b2 mRNA COVID-19 (booster) vaccine dose. [file 21-2330-Techapp-s1.pdf]

Article DOI: https://doi.org/10.3201/eid2807.212330

# Self-Reported and Physiologic Reactions to Third BNT162b2 mRNA COVID-19 (Booster) Vaccine Dose

## Appendix Part A: Study Protocol

### Background

Vaccination is widely accepted as the most prominent measure in the fight against COVID-19, posing the greatest hope for ending this major global health pandemic and related economic crisis (*1*). Consequently, an unprecedented international effort by private and public institutions alike was directed at accelerating the traditionally lengthy vaccine-development process (*2–4*).

On 2 December 2020, less than a year from the pandemic outbreak, the first vaccine, BNT162b2 mRNA (Pfizer-BioNTech), was granted an Emergency Use Authorization (EUA) by the UK Medicines and Healthcare Products Regulatory Agency (MHRA) (*5*). This initial authorization was followed by rapid authorizations for emergency use in several countries, with the U.S. Food and Drug Administration (FDA) among the first to do so (*6*).

Safety data from a randomized controlled trial suggests a favorable safety profile for the BNT162b2 vaccine (*7*). Specifically, the local and systemic self-reported reactions during the first 7 days after vaccination were mainly mild to moderate, with a median onset of 0–2 days after vaccine administration and a median duration of 1–2 days. The most frequently reported reactions were fatigue, headache, muscle pain, chills, joint pain, and fever (*7*). The incidence of serious adverse events was low and was similar between vaccine- and placebo-treated participants. The safety of the new vaccine over a median of 2 months post-vaccination was similar to that of other viral vaccines. A considerable fraction of the participants did not report any reaction or adverse event. Likewise, several other vaccine candidates, including ChAdOx1 nCoV-19 (Oxford/AstraZeneca) and

mRNA-1273 (Moderna), received EUAs following similar encouraging safety results by randomized controlled trials (*8–10*).

The SARS-CoV-2 Delta variant (also termed variant B.1.617.2) was discovered in October 2020, in India, and was designated as a variant of concern by the World Health Organization (WHO) in May 2021 (*11–13*). The rapid increase in hospitalizations in Israel associated with the Delta-driven COVID-19 resurgence, and the imminent risk for hospital overcrowding, led the Israeli government to initialize on July 30, 2021, an unparalleled, pro-active, national third (booster) vaccine shot campaign, offering the BNT162b2 mRNA COVID-19 vaccine to persons over the age of 60. On August 13, 2021, the booster campaign was expanded to include those over 50 years of age, reaching 63% third-dose coverage among the eligible population within only 26 days (*14–17*). Two weeks later, on August 29, 2021, the campaign was expanded to include all persons 16+ of age, demanding only that 5 months have passed since the receipt of the second dose, reaching 40% third-dose coverage among the eligible population under 50 years of age, within 16 days (*18,19*).

Currently, limited information is available on the safety of a BNT162b2 third dose (*20,21*), with such a booster vaccine yet to be authorized by the U.S. Food and Drug Administration (FDA) to the general population (*22*). While recent evidence shows that a third BNT162b2 dose in immunocompromised persons has a favorable safety profile (*21,23*), the safety of a third (booster) dose in the general population has not yet been fully established.

## Methods

### Study Design

In this study we will analyze data that was already collected and will be collected as part of the PerMed study (*24*). Participants in the PerMed study are recruited for a period of 2 years, during which they are equipped with a Garmin Vivosmart 4 smartwatches and are asked to wear them as much as they could. In addition, participants install 2 applications on their mobile phones: an application that passively collects data from the smartwatch and a dedicated mobile application that enables participants to fill a

daily questionnaire and to report their vaccine date and specific hour. In this study, we will consider for each participant, the 7-days period before any vaccination dose as the baseline period.

### Participants

The inclusion criteria for the PerMed study includes those aged >18 years. Persons who are not eligible to give and sign a consent form of their free are excluded. In this study, we will analyze the data of participants aged 18 years and above, who reported receiving at least 1 dose of the BNT162b2 mRNA COVID-19 vaccine after joining the PerMed study. To recruit participants and ensure they complete all the study's requirements, we will hire a professional survey company. Potential participants will be recruited through advertisements in social media, online banners, and word-of-mouth. The survey company is responsible for guaranteeing the participants meet the study's requirements, in particular, that the questionnaires are filled daily, ensuring the smartwatches are charged constantly and worn properly, and assisting participants resolve technical problems.

### Study Procedures

Before participation in the study, all participants will be advised orally and in writing about the nature of the experiments and give written, informed consent. At this time, participants will be asked to complete an enrollment questionnaire that includes demographic information and health status. In addition, participants will be asked to install 2 applications on their mobile phones: an application that passively collects data from the smartwatch and the PerMed application, which enables participants to fill in the daily questionnaires. Participants will be given instructions regarding the self-reported symptoms questionnaires and how to operate the smartwatch, which they will wear as much as they can.

### Enrollment Questionnaire

All participants will fill a 1-time enrollment questionnaire that includes demographic questions and questions about the participant's health condition in general. Specifically, the questionnaire will include the following: age, sex, height, weight and underlying medical conditions (Listed in Table 1, main text). Other questions such as

name, address, phone and email will be recorded and used by the survey company to contact the participants. The answers will be filled-in directly by the survey company to the study's secured dashboard.

#### Monitoring Device

Participants will be equipped with Garmin Vivosmart 4 smart fitness trackers. Among other features, the smartwatch provides all-day heart rate and heart rate variability and during-night blood oxygen saturation level tracking capabilities (*25*).

The optical wrist heart rate (HR) monitor of the smartwatch is designed to continuously monitor a user's heart rate. The frequency at which heart rate is measured varies and may depend on the level of activity of the user: when the user starts an activity, the optical HR monitor's measurement frequency increases.

Since heart rate variability (HRV) is not easily accessible through Garmin's application programming interface (API), we use Garmin's stress level instead, which is calculated based on HRV. Specifically, the device uses heart rate data to determine the interval between each heartbeat. The variable length of time between each heartbeat is regulated by the body's autonomic nervous system. Less variability between beats correlates with higher stress levels, whereas an increase in variability indicates less stress (*26*). A similar relationship between HRV and stress was also seen in (*27,28*).

The Pulse Ox monitor of the smartwatch uses a combination of red and infrared lights with sensors on the back of the device to estimate the percentage of oxygenated blood (peripheral oxygen saturation). The Pulse Ox monitor is activated each day at a fixed time for a period of 4 hours (the default is 2:00 AM–6:00 AM).

Examining the data collected in our study, we identified an HR sample roughly every 15 seconds, an HRV sample every 180 seconds, and an blood oxygen saturation level sample every 60 seconds.

While the Garmin smartwatch provides state-of-the-art wrist monitoring, it is not a medical-grade device, and some readings may be inaccurate under certain circumstances, depending on factors such as the fit of the device and the type and intensity of the activity undertaken by a participant (*29–31*).

### Vaccination Questionnaire

The vaccination questionnaire we will use includes the following question:

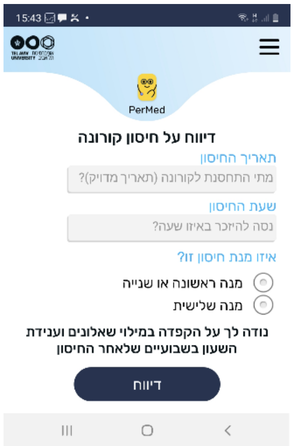


COVID-19 vaccination – date, time, and dose number.

### Daily Questionnaires

All participants will complete the daily self-reported questionnaire in a dedicated application (the PerMed mobile application). The daily questionnaire we will use includes the following questions:

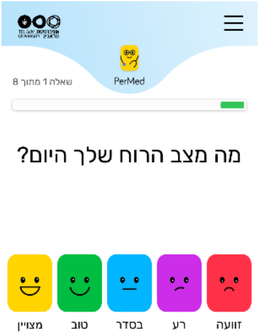


How is your mood today? • Awful (−2)• Bad (−1)• OK (0)• Good (1)• Excellent (2)

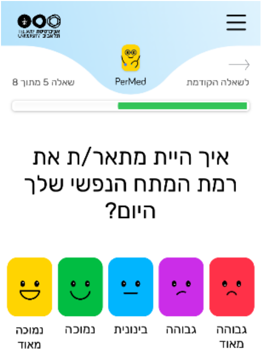


How would you describe the level of your stress during the last day?• Very Low (−2)• Low (−1)• Medium (0)• High (1)• Very high (2)

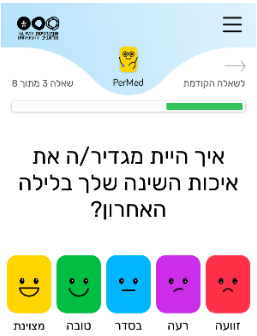


How would you define your last night sleep quality?• Awful (−2)• Bad (−1)• OK (0)• Good (1)• Excellent (2)

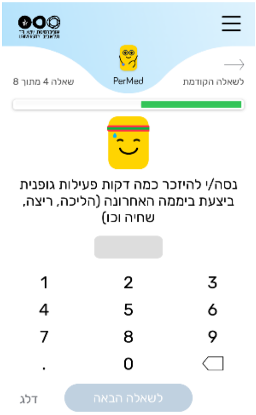


Try to remember how many minutes of sports activity you performed on the last day?

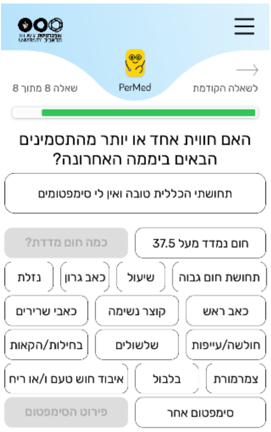


Have you experienced one or more of the following symptoms in the last 24 hours?• My general feeling is good, and I have no symptoms• Heat measured above 37.5• Cough• Sore throat• Runny nose• Headache• Shortness of breath• Muscle aches• Weakness / fatigue• Diarrhea• Nausea / vomiting• Chills• Confusion• Loss of sense of taste / smell• Another symptom.

### Data Storage

Data collected from the mobile phone application and from the smartwatches will be stored on a secure server within Tel Aviv University facilities. The server runs a CentOS operating system and is located in Software Engineering Building at Tel Aviv University. This server is protected behind the university's firewall and is not connected to external networks. In addition, a secure connection through an SSL protocol and a

trusted certificate will be obtained for the transfer of information from the mobile phone application into the secured server.

Access will be restricted to investigators in the study. The information from the mobile application will be stored in a structured manner on the secured server without any explicitly identifying information (name, ID number, email). Each participant will be assigned a coded participant number that will be used to identify the subject in the database. The code with the identified information will be stored in an encrypted form on a separate secured server that only the research manager will have access to. Access to all servers is restricted with username and password.

All (non-digital) questionnaires and signed informed consent documents will be stored in a secured cabinet in Tel Aviv University, to which only the research manager and the principal investigators will have access. No data collected as part of the study will be added to persons' medical charts.

### Data Processing

We will perform several preprocessing steps. Concerning the daily questionnaires, in cases where participants will fill in the daily questionnaire more than once on a given day, only the last entry for that day will be considered, as it is reasoned that the last one likely best represented the entire day. Self-reported symptoms that are entered as the free text will be manually categorized. With regard to the smartwatch physiologic indicators, data will first be aggregated per hour (by taking the mean value). Then, to impute missing values, we will perform a linear interpolation. Finally, data will be smoothed by calculating the moving average value using a 5-hour sliding window.

### Data Analysis

For each participant, we defined the 7-day period before vaccination as a baseline. First, for the period of 48 hours from vaccination, we will calculate the percentage of participants who reported new local or systemic reactions compared with their baseline period (i.e., the last questionnaire each participant filled during the baseline period). For each reaction, a 90% confidence interval will be calculated, assuming a β distribution, with parameter $\alpha$ corresponding to the number of participants reporting that reaction plus one (i.e., "successes"), and parameter $\beta$ corresponding to the number of participants who

did not report that reaction plus one (i.e., "failures"). To determine the statistical significance of differences between the first and third doses and between the second and third doses as reflected by the extent of reported reactions, we will use a 2-proportion Z-test.

Next, we will calculate the changes in well-being indicators reported post-vaccination compared with those reported during the baseline period. Specifically, for each indicator and each participant, we will calculate the difference between the value in each of the 3 days post-vaccination and the corresponding value in the baseline period (i.e., the last questionnaire filled during the baseline period). Then, for each indicator and each of the 3 days post vaccination, we will calculate the mean difference value over all participants and the associated 90% confidence interval.

Finally, we will compare the changes in smartwatch physiologic indicators over the 7 days (168 hours) post vaccination with those of the baseline period. To do so, we will perform the following steps. First, for each participant and each hour during the 7 days post vaccination, we will calculate the difference between that hour's indicator value and that of the corresponding hour in the baseline period (keeping the same day of the week and same hour during the day). Then, we will calculate the mean difference value for each hour over all participants, as well as the 90% confidence interval, corresponding to a significance level of 0.05 in a 1-sided t-test. To determine the statistical significance of differences between the first and third doses and between the second and third doses as reflected by changes in smartwatch indicators during the 48 hours post-vaccination, we will use a 2-sample t-test with unequal variance.

#### Potential Risks and Risk Management

No specific risks arising from the smartwatches are expected, as the device is already commercialized with no known adverse reactions. The main risk in this study is the leakage of private data which we intend to manage as we describe in the following section.

#### Privacy/Confidentiality

Results from this study will be handled at an aggregated level. Individual data records will remain confidential and will not be published or shared with any third party.

Signed and dated informed consent forms, as well as data recording sheets (e.g., case report forms) will be stored in locked cabinets during the study and following its completion. A file containing the personal details of the participants will be coded to help preserve confidentiality and will be separated from all other data collected throughout the study. This file will be kept by the principal investigator. Data will be stored on computers in password-protected files.

The data obtained from the smartwatch used in this study will be linked to a coded participant number. The smartwatch does not include a global positioning system. Data collected by the PerMed application will arrive directly to PerMed back-end servers and will be stored securely.

## Appendix Part B: Additional Results

### Changes in Objective Physiologic Indicators Measured through the Smartwatch following the Three Vaccine Doses

Changes in objective physiologic indicators observed during the first 2 days after the second and third vaccine doses are similar, and considerably greater than those observed following the first dose.

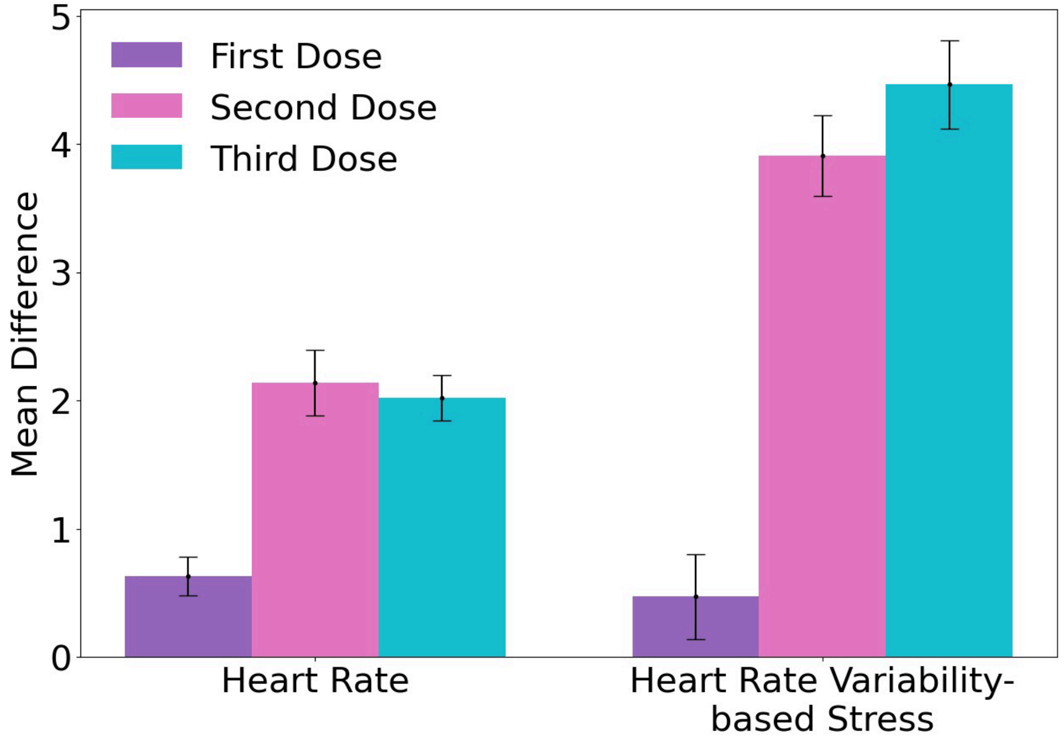


**Appendix Figure 1.** Changes in objective physiologic indicators measured through the smartwatch during the first 2 days after vaccine. Mean difference in smartwatch-recorded heart rate and heart rate variability-based following the first, second and third dose, compared with their baseline levels. Changes in objective physiologic indicators were calculated by subtracting the baseline values from the mean value of the first 2 days following the vaccine dose. Error bars represent 90% confidence intervals.

**Changes in reported well-being indicators – stratification by age group**

Changes in well-being observed during the first 2 days after the third vaccine dose were found to be higher for participants younger than 50 years compared with those between 50 and 65 years, and consequently higher than those older than 65 years, with the exception of reported stress level (Appendix Figure 2).

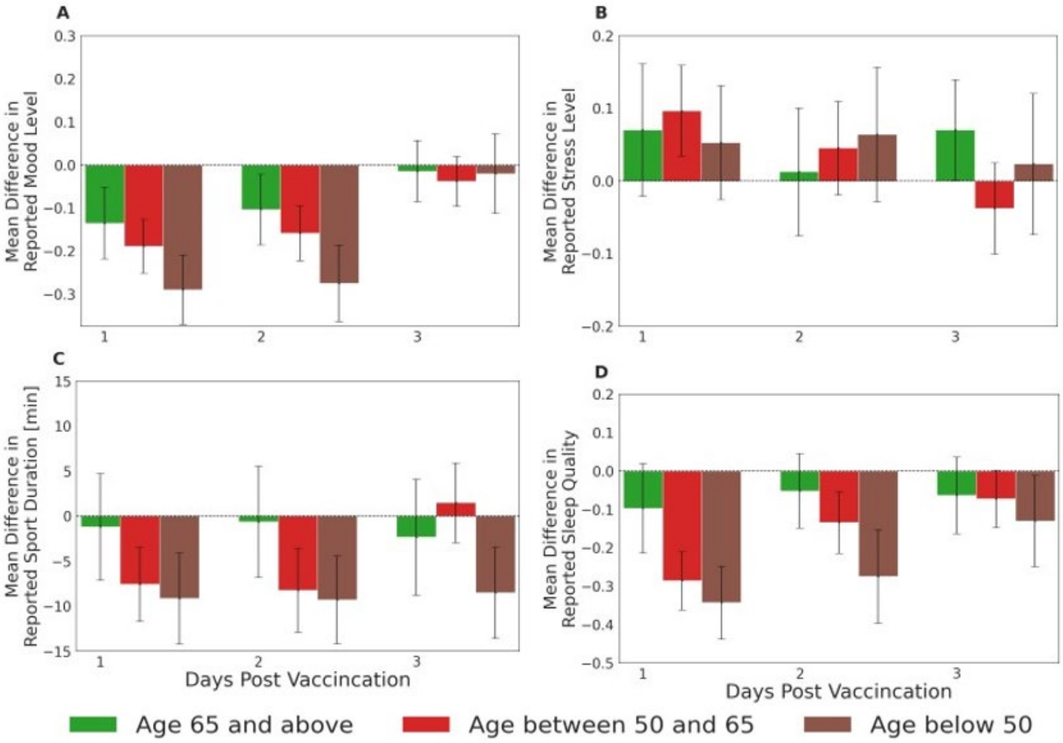


**Appendix Figure 2.** Changes in well-being indicators reported by participants through the mobile application stratified by age group. (A) mood level, measured on a 1-to-5 Likert scale. (B) Stress level, measured on a 1-to-5 Likert scale. (C) Sport duration, measured in minutes. (D) Sleep quality, measured on a 1-to-5 Likert scale. Changes in well-being indicators were calculated by subtracting the baseline values from the daily values. Error bars represent 90% confidence intervals. Horizontal dashed lines represent no change compared with baseline levels.

**Changes in Reported Well-Being Indicators – Stratification by Sex**

Changes in well-being observed during the first 2 days after the third vaccine dose were found to be similar for males and females (Appendix Figure 3).

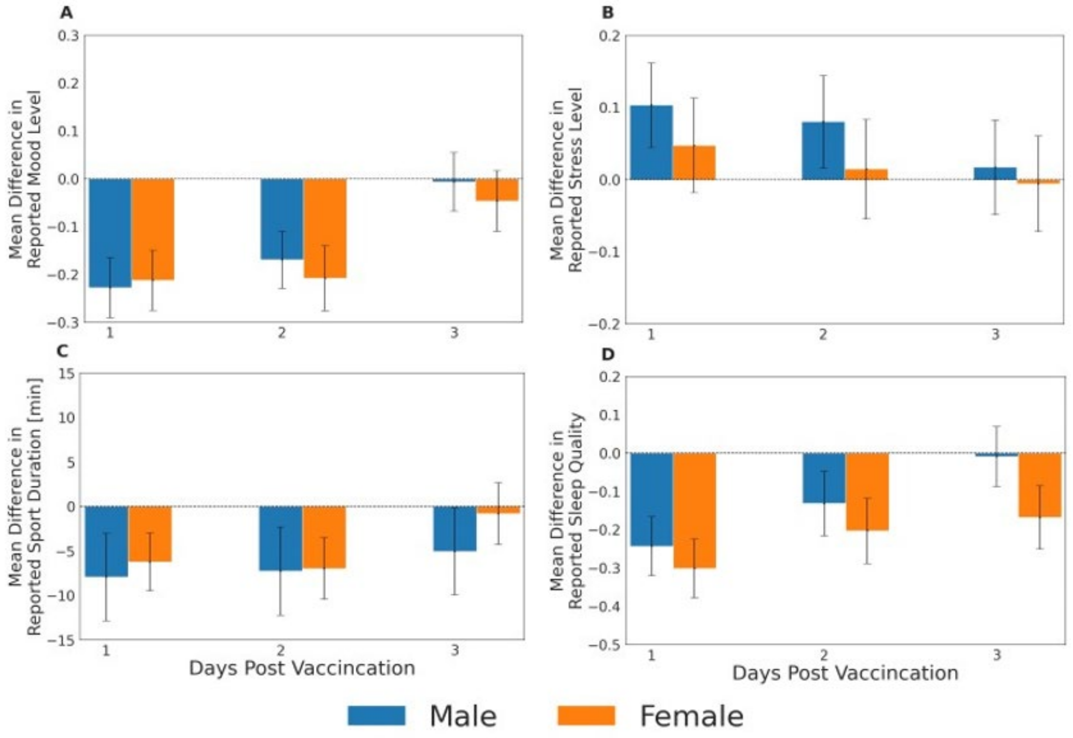


**Appendix Figure 3.** Changes in well-being indicators reported by participants through the mobile application stratified by sex. (A) mood level, measured on a 1-to-5 Likert scale. (B) Stress level, measured on a 1-to-5 Likert scale. (C) Sport duration, measured in minutes. (D) Sleep quality, measured on a 1-to-5 Likert scale. Changes in well-being indicators were calculated by subtracting the baseline values from the daily values. Error bars represent 90% confidence intervals. Horizontal dashed lines represent no change compared with baseline levels.

**Changes in reported well-being indicators – stratification by underlying medical condition**

Changes in mood level and sleep quality observed during the first 2 days after the third vaccine dose were found to be higher for participants without underlying medical conditions compared with those with underlying medical condition (Appendix Figure 4).

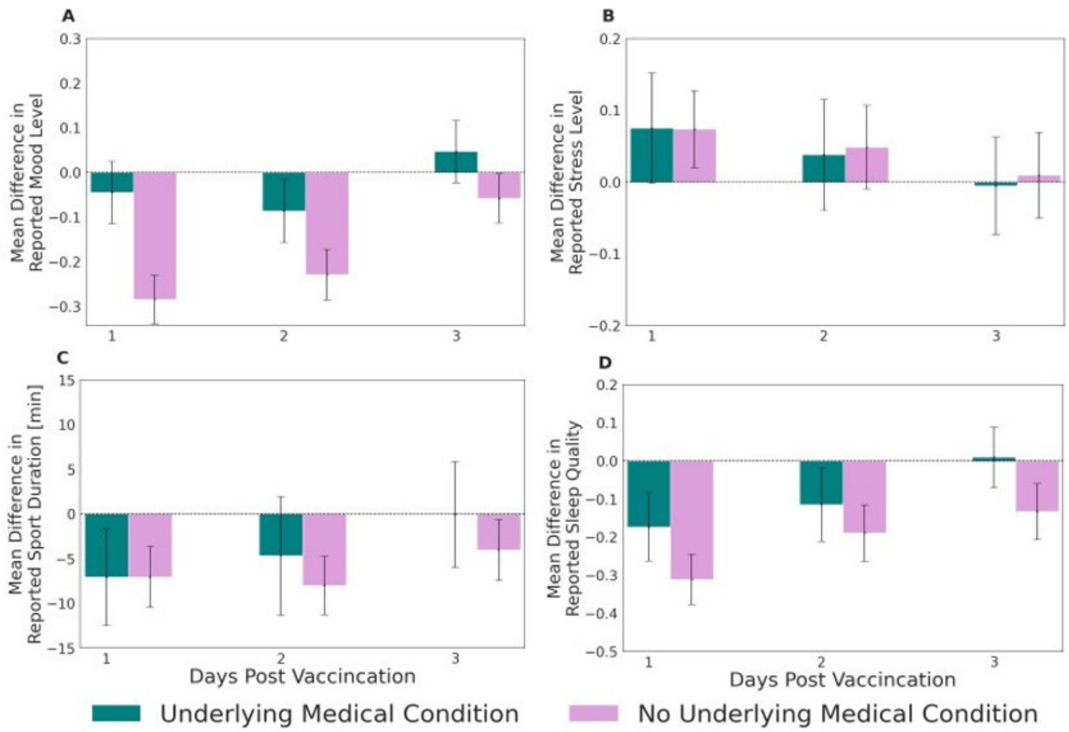


**Appendix Figure 4.** Changes in well-being indicators reported by participants through the mobile application stratified by underlying medical conditions. (A) mood level, measured on a 1-to-5 Likert scale. (B) Stress level, measured on a 1-to-5 Likert scale. (C) Sport duration, measured in minutes. (D) Sleep quality, measured on a 1-to-5 Likert scale. Changes in well-being indicators were calculated by subtracting the baseline values from the daily values. Error bars represent 90% confidence intervals. Horizontal dashed lines represent no change compared with baseline levels.

**Changes in Physiologic Indicators – Stratification by Age Group**

Changes in physiologic indicators after the third vaccine dose stratified by age group were consistent with those observed in the general population (considerable changes during the first 2 days after vaccine administration that faded nearly entirely after 3 days). These changes were found to be higher for participants younger than 50 years compared with those between 50 and 65 years, and consequently higher than those older than 65 years (Appendix Figure 5).

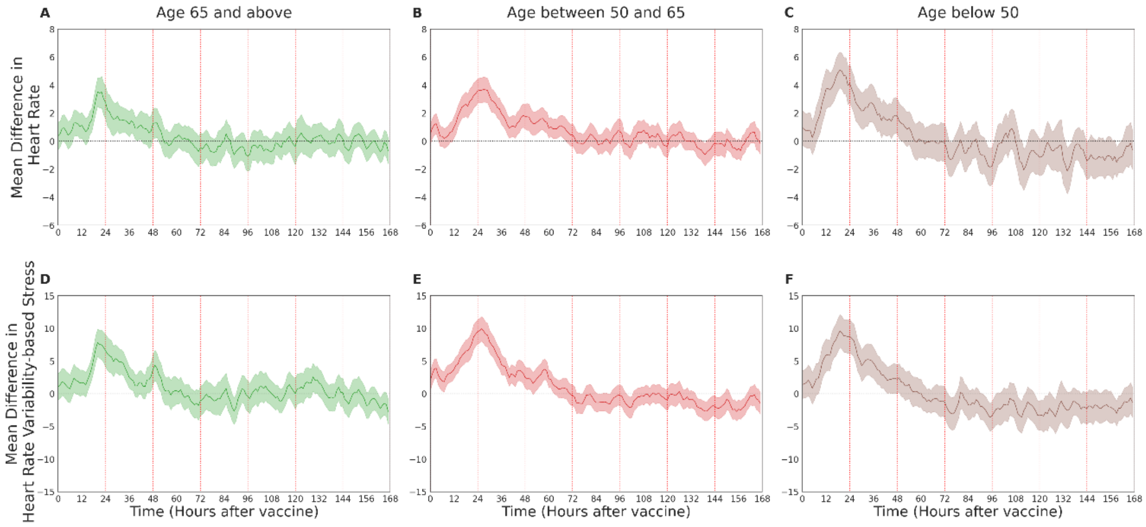


**Appendix Figure 5.** Changes in physiologic indicators measured through the smartwatch stratified by age groups. Mean difference in heart rate and heart rate variability-based stress indicators following the third dose, recorded by a smartwatch, compared with their baseline levels: (**A** and **B**) heart rate, (**C** and **D**) heart rate variability-based stress. Mean values are depicted as solid lines; 90% confidence intervals are presented as shaded regions. The horizontal dashed line represents no change compared with the baseline levels, and vertical lines represent 24-hour periods.

**Changes in Physiologic Indicators – Stratification by Sex**

Changes in physiologic indicators after the third vaccine dose stratified by sex were consistent with those observed in the general population (considerable changes during the first 2 days after vaccine administration that faded nearly entirely after 3 days). These changes were found to be higher for females compared with males (Appendix Figure 6).

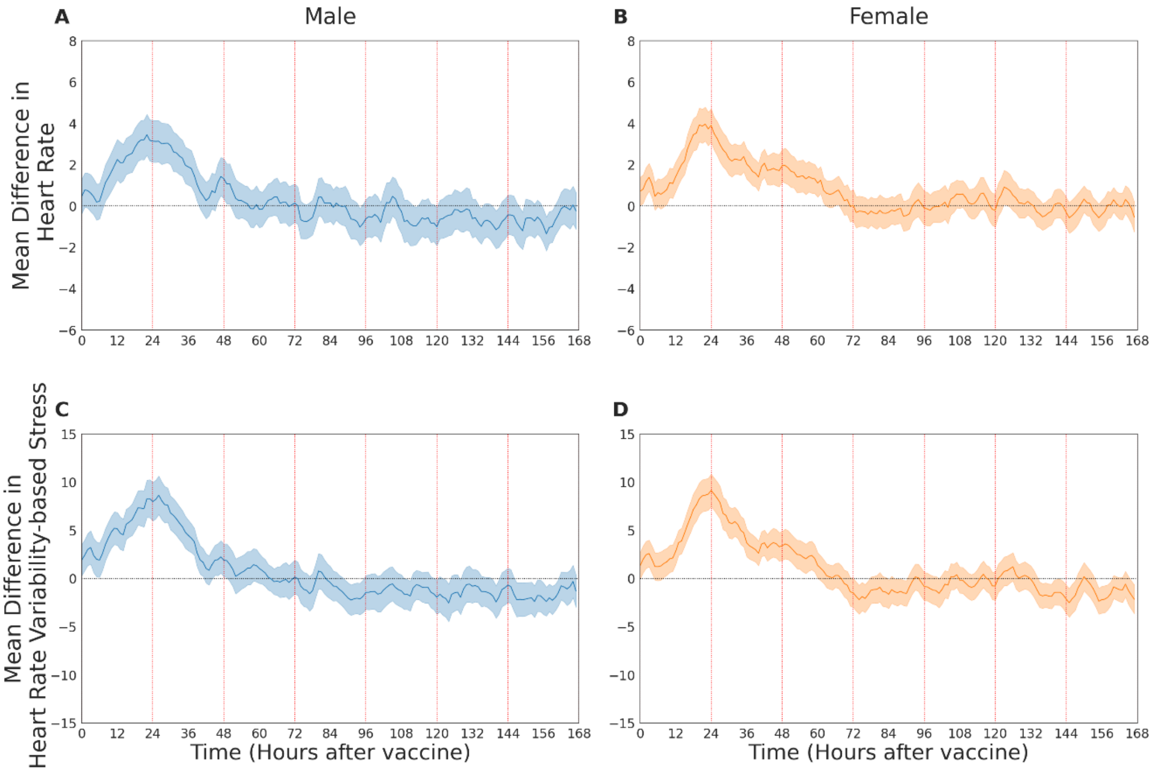


**Appendix Figure 6.** Changes in physiologic indicators measured through the smartwatch stratified by sex. Mean difference in heart rate and heart rate variability-based stress indicators following the third dose, recorded by a smartwatch, compared with their baseline levels: (**A** and **B**) heart rate, (**C** and **D**) heart rate variability-based stress. Mean values are depicted as solid lines; 90% confidence intervals are presented as shaded regions. The horizontal dashed line represents no change compared with the baseline levels, and vertical lines represent 24-hour periods.

**Changes in Physiologic Indicators – Stratification by Underlying Medical Condition**

Changes in physiologic indicators after the third vaccine dose stratified by underlying medical condition were consistent with those observed in the general population (considerable changes during the first 2 days after vaccine administration that faded nearly entirely after 3 days). These changes were found to be higher for participants without underlying medical conditions compared with those with underlying medical condition (Appendix Figure 7).

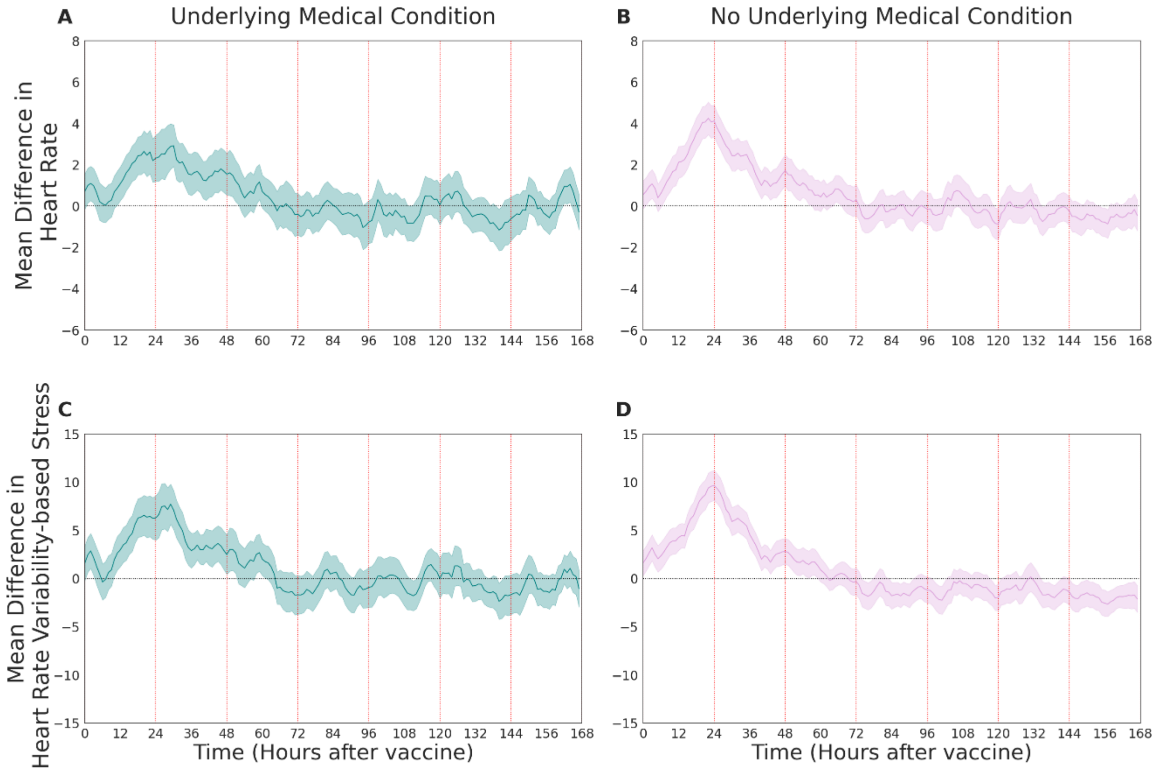


**Appendix Figure 7.** Changes in physiologic indicators measured through the smartwatch stratified by underlying medical conditions. Mean difference in heart rate and heart rate variability-based stress indicators following the third dose, recorded by a smartwatch, compared with their baseline levels: (A and B) heart rate and (C and D) heart rate variability-based stress. Mean values are depicted as solid lines; 90% confidence intervals are presented as shaded regions. The horizontal dashed line represents no change compared with the baseline levels, and vertical lines represent 24-hour periods.

**Thirty Days Analysis for Self-Reported Local and Systemic Reactions after the Third Dose**

We observe a sharp decline in reported local and systemic reactions following 3 days after the third vaccination dose, and nearly a complete halt within 30 days post-vaccination (Appendix Figure 8). Fatigue and headache were the most frequent reactions reported and lasted longer than the other reported reactions.

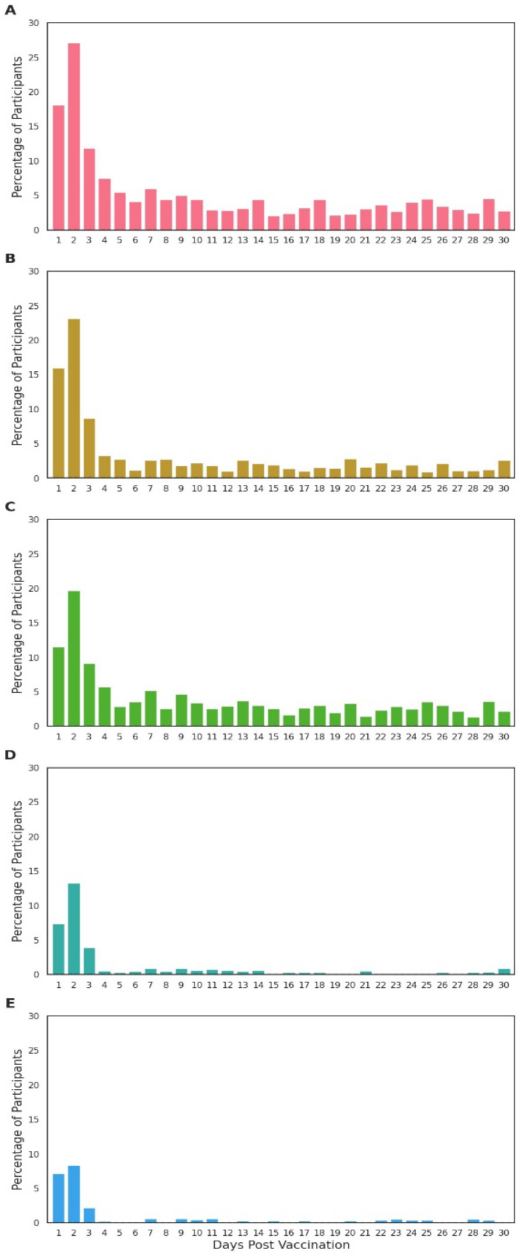


**Appendix Figure 8.** Most frequent local and systemic reactions reported by participants through the mobile application after the third dose. (A) fatigue, (B) muscle pain, (C) headache, (D) fever, and (E) chills.

### Pairwise Analysis of Doses

A considerable number of participants joined our study after receiving the first or second dose. Thus, in our main analyses, we compared the third dose to the first or second dose by using statistical significance tests for comparing the means of 2 partially overlapping samples with unequal variance. To further support the results of our main analyses, we also conducted an analysis where we examined changes in reactions for the subgroups of participants who reported receiving all 3 doses. To determine the statistical significance of differences in the proportions of participants' self-reported reactions between doses, we used McNemar tests (Appendix Table 1). To determine the statistical significance of differences in the change in smartwatch measurements between doses we used paired t-tests (Appendix Table 2).

**Appendix Table 1.** Self-reported reactions (N = 53)

| Compared doses | % participants who reported no reaction after vaccination | p value |
|---|---|---|
| First and third | First dose: 84.91%, third dose: 64.51% | <0.001 |
| Second and third | Second dose: 75.47%, third dose: 64.51% | <0.01 |

**Appendix Table 2.** Change in smartwatch measurements (N = 69)

| Measure | Compared doses | Mean difference in measure in the 48 h postvaccination | p value |
|---|---|---|---|
| Heart rate | First and third | First dose: 0.521, third dose: 2.168 | <0.05 |
| | Second and third | Second dose: 1.617, third dose: 2.168 | 0.405 |
| Heart rate variability | First and third | First dose: 1.298, third dose: 6.204 | <0.01 |
| | Second and third | Second dose: 4.343, third dose: 6.204 | 0.239 |

### Calculation of Required Sample Size

The primary goal of the study was to compare reactions following the third dose to those observed in the first and second doses. Particularly, we wanted to know whether reactions following the third dose will be greater than those observed following the second dose. The logic for this notion is the expectation that reactions following primary exposure are typically milder than those following subsequent exposure (namely second or booster dose). Thus, to determine the required sample size, we first identified the five most prevalent systemic reactions observed during the Pfizer clinical trial: headache, fatigue, fever, chills, and muscle pain. Based on those trials, the frequency of these reactions ranged between 16%–59% in persons 16–55, and 11%–51% in persons >55 (*7*). We considered a standard statistical power of >80% for a scenario where reactions are

more prevalent at least 10% than those observed in the clinical trials. We conservatively assumed a non-repeated framework (i.e., different participants received the third dose than those who received the first and second doses). Taken together, we used a Z-test to evaluate the difference in the two population proportions. Under the standard assumptions of $\alpha = 0.05$ , $\beta = 1 - \pi = 0.8$, and the formula $(Z_{\alpha} + Z_{\beta})^2 \times (p_1(1 - p_1) + p_2(1 - p_2))/(p_1 - p_2)^2$, we derived a required n = 203–282 in persons 18–55 (which correspond to 16%–59%) namely >282, and 164–302 (which corresponds to 11%–51%) namely n > 302 in persons >55. To conclude, the actual number of participants in our study is considerably larger than the one required to ensure statistical significance.

The primary goal of the study was to compare reactions following the third dose to those

**References**


1. World Health Organization. Draft landscape and tracker of COVID-19 candidate vaccines [cited 2022 Apr 13]. https://www.who.int/publications/m/item/draft-landscape-of-covid-19-candidate-vaccine

2. Sharma O, Sultan AA, Ding H, Triggle CR. A review of the progress and challenges of developing a vaccine for COVID-19. Front Immunol. 2020;11:585354. PubMed https://doi.org/10.3389/fimmu.2020.585354

3. Parker EP, Shrotri M, Kampmann B. Keeping track of the SARS-CoV-2 vaccine pipeline. Nat Rev Immunol. 2020;20:650. PubMed https://doi.org/10.1038/s41577-020-00455-1

4. Krammer F. SARS-CoV-2 vaccines in development. Nature. 2020;586:516–27. PubMed https://doi.org/10.1038/s41586-020-2798-3

5. MHRA guidance on coronavirus (COVID-19) - GOV.UK [cited 2022 Apr 13]. https://www.gov.uk/government/collections/mhra-guidance-on-coronavirus-covid-19

6. Food and Drug Administration. FDA takes key action in fight against COVID-19 by issuing emergency use authorization for first COVID-19 vaccine [cited 2022 Apr 13]. https://www.fda.gov/news-events/press-announcements/fda-takes-key-action-fight-against-covid-19-issuing-emergency-use-authorization-first-covid-19

7. Polack FP, Thomas SJ, Kitchin N, Absalon J, Gurtman A, Lockhart S, et al.; C4591001 Clinical Trial Group. Safety and efficacy of the BNT162b2 mRNA COVID-19 vaccine. N Engl J Med. 2020;383:2603–15. PubMed https://doi.org/10.1056/NEJMoa2034577

8. World Health Organization. Background document on mRNA vaccine BNT162b2 (Pfizer-BioNTech) against COVID-19 [cited 2022 Apr 13]. https://www.who.int/publications/i/item/background-document-on-mrna-vaccine-bnt162b2-(pfizer-biontech)-against-covid-19

9. Baden LR, El Sahly HM, Essink B, Kotloff K, Frey S, Novak R, et al.; COVE Study Group. Efficacy and safety of the mRNA-1273 SARS-CoV-2 vaccine. N Engl J Med. 2021;384:403–16. PubMed https://doi.org/10.1056/NEJMoa2035389

10. Voysey M, Clemens SA, Madhi SA, Weckx LY, Folegatti PM, Aley PK, et al.; Oxford COVID Vaccine Trial Group. Safety and efficacy of the ChAdOx1 nCoV-19 vaccine (AZD1222) against SARS-CoV-2: an interim analysis of four randomised controlled trials in Brazil, South Africa, and the UK. Lancet. 2021;397:99–111. PubMed https://doi.org/10.1016/S0140-6736(20)32661-1

11. Kupferschmidt K, Wadman M. Delta variant triggers new phase in the pandemic. Science. 2021;372:1375–6. https://doi.org/10.1126/science.372.6549.1375

12. World Health Organization. Tracking SARS-CoV-2 variants [cited 2022 Apr 13]. https://www.who.int/en/activities/tracking-SARS-CoV-2-variants

13. Mlcochova P, Kemp SA, Dhar MS, Papa G, Meng B, Ferreira IA, et al.; Indian SARS-CoV-2 Genomics Consortium (INSACOG); Genotype to Phenotype Japan (G2P-Japan) Consortium; CITIID-NIHR BioResource COVID-19 Collaboration. SARS-CoV-2 B.1.617.2 Delta variant replication and immune evasion. Nature. 2021;599:114–9. PubMed https://doi.org/10.1038/s41586-021-03944-y

14. Ministry of Health. The Ministry of Health Director General Has Approved the Recommendation to Administer a Third Vaccine Dose to 50-Year-Olds and Older and to Other Populations | Ministry of Health [cited 2022Apr 13]. https://www.gov.il/en/departments/news/13082021-01

15. Ministry of Health. The Vaccination Advisory Committee Presented Data and Recommended the Administration of a Third Dose to Older Adults [cited 2022Apr 13]. https://www.gov.il/en/departments/news/29072021-04

16. Prime Minister's Office. Statement by PM Bennett on Third Dose of the COVID Vaccine to Israeli Citizens over the Age of 60 [cited 2022 Apr 13]. https://www.gov.il/en/Departments/news/event_press29071

17. Wadman M. Israel's grim warning: Delta can overwhelm shots. Science. 2021;373:838–9. PubMed https://doi.org/10.1126/science.373.6557.838

18. Ministry of Health. The Vaccination Policy and the New Green Pass that will Take Effect Soon [cited 2022 Apr 13]. https://www.gov.il/en/departments/news/29082021-01

19. Centers for Disease Control and Prevention. COVID-19 Datasets- Government Data [cited 2022 Apr 13]. https://data.cdc.gov

20. Centers for Disease Control and Prevention. Joint Statement from HHS Public Health and medical experts on COVID-19 booster shots [2022 Apr 13]. https://www.cdc.gov/media/releases/2021/s0818-covid-19-booster-shots.html

21. Centers for Disease Control and Prevention. COVID-19 Vaccines for Moderately to Severely Immunocompromised People [cited 2022 Apr 13]. https://www.cdc.gov/coronavirus/2019-ncov/vaccines/recommendations/immuno.html?s_cid=10483:immunocompromised%20and%20covid%20vaccine:sem.ga:p:RG:GM:gen:PTN:FY21

22. Food and Drug Administration. Joint statement from HHS public health and medical experts on COVID-19 booster shots [cited 2022 Apr 22]. https://www.hhs.gov/about/news/2021/08/18/joint-statement-hhs-public-health-and-medical-experts-covid-19-booster-shots.html

23. Kamar N, Abravanel F, Marion O, Couat C, Izopet J, Del Bello A. Three doses of an mRNA Covid-19 vaccine in solid-organ transplant recipients. N Engl J Med. 2021;385:661–2. PubMed https://doi.org/10.1056/NEJMc2108861

24. Oved S, Mofaz M, Lan A, Einat H, Kronfeld-Schor N, Yamin D, et al. Differential effects of COVID-19 lockdowns on well-being: interaction between age, gender and chronotype. J R Soc Interface. 2021;18:20210078. PubMed https://doi.org/10.1098/rsif.2021.0078

25. Garmin. VÍVOSMART® 4 Owner's Manual. 2018 [cited 2022 Apr 13]. https://www8.garmin.com/manuals/webhelp/vivosmart4/EN-US/vivosmart_4_OM_EN-US.pdf

26. Garmin. What Is the Stress Level Feature on My Garmin Watch? [cited 2022 Apr 13]. https://support.garmin.com

27. Kim H-G, Cheon E-J, Bai D-S, Lee YH, Koo B-H. Stress and heart rate variability: a meta-analysis and review of the literature. Psychiatry Investig. 2018;15:235–45. PubMedhttps://doi.org/10.30773/pi.2017.08.17

28. Pereira T, Almeida PR, Cunha JP, Aguiar A. Heart rate variability metrics for fine-grained stress level assessment. Comput Methods Programs Biomed. 2017;148:71–80. PubMed https://doi.org/10.1016/j.cmpb.2017.06.018

29. Reddy RK, Pooni R, Zaharieva DP, Senf B, El Youssef J, Dassau E, et al. Accuracy of wrist-worn activity monitors during common daily physical activities and types of structured exercise: evaluation study. JMIR Mhealth Uhealth. 2018;6:e10338. PubMed https://doi.org/10.2196/10338

30. Bent B, Goldstein BA, Kibbe WA, Dunn JP. Investigating sources of inaccuracy in wearable optical heart rate sensors. NPJ Digit Med. 2020;3:18. PubMed https://doi.org/10.1038/s41746-020-0226-6

31. Garmin. Accuracy [cited 2022 Apr 13]. https://www.garmin.com/en-US
